# Genome-Wide Identification of WRKY Genes in *Artemisia annua*: Characterization of a Putative Ortholog of *AtWRKY40*

**DOI:** 10.3390/plants9121669

**Published:** 2020-11-28

**Authors:** Angelo De Paolis, Sofia Caretto, Angela Quarta, Gian-Pietro Di Sansebastiano, Irene Sbrocca, Giovanni Mita, Giovanna Frugis

**Affiliations:** 1 Istituto di Scienze delle Produzioni Alimentari (ISPA), Consiglio Nazionale delle Ricerche (CNR), Via Monteroni, 73100 Lecce, Italy; angela.quarta@libero.it (A.Q.); giovanni.mita@ispa.cnr.it (G.M.); 2 DiSTeBA (Dipartimento di Scienze e Tecnologie Biologiche ed Ambientali), University of Salento, Campus ECOTEKNE, 73100 Lecce, Italy; gp.disansebastiano@unisalento.it; 3 Istituto di Biologia e Biotecnologia Agraria (IBBA), Consiglio Nazionale delle Ricerche (CNR), Via Salaria, Km 29.300, 00015 Rome, Italy; irene.sbrocca@gmail.com (I.S.); giovanna.frugis@cnr.it (G.F.)

**Keywords:** *Artemisia annua*, WRKY transcription factor, genome-wide identification, phylogenetic analysis, expression analysis

## Abstract

*Artemisia annua* L. is well-known as the plant source of artemisinin, a sesquiterpene lactone with effective antimalarial activity. Here, a putative ortholog of the *Arabidopsis thaliana* WRKY40 transcription factor (TF) was isolated via reverse transcription-polymerase chain reaction and rapid amplification of cDNA ends in *A. annua* and named *AaWRKY40*. A putative nuclear localization domain was identified in silico and experimentally confirmed by using protoplasts of *A. annua* transiently transformed with *AaWRKY40-GFP*. A genome-wide analysis identified 122 *WRKY* genes in *A. annua*, and a manually curated database was obtained. The deduced proteins were categorized into the major WRKY groups, with group IIa containing eight WRKY members including AaWRKY40. Protein motifs, gene structure, and promoter regions of group IIa WRKY TFs of *A. annua* were characterized. The promoter region of *AaWRKY* group IIa genes contained several abiotic stress *cis*-acting regulatory elements, among which a highly conserved W-box motif was identified. Expression analysis of *AaWRKY40* compared to *AaWRKY1* in *A. annua* cell cultures treated with methyl jasmonate known to enhance artemisinin production, suggested a possible involvement of *AaWRKY40* in terpenoid metabolism. Further investigation is necessary to study the role of AaWRKY40 and possible interactions with other TFs in *A. annua*.

## 1. Introduction

Most plant biosynthetic genes related to biotic and abiotic stress responses and/or involved in the production of secondary metabolites are under the control of different transcription factor (TF) families [1,2] and the cross-talk between the different members of each family amplifies the complexity of possible transcriptional regulatory roles [3].

WRKY proteins are a large family of plant TFs involved in the regulation of several physiological processes including development, senescence, and immune response. Since the first reports on the cloning of *WRKY* cDNAs from sweet potato, oat, parsley, and Arabidopsis [4,5,6], several members of the *WRKY* family have been described [7]. WRKY TFs have been demonstrated to share a conserved 60 amino acid sequence containing the highly conserved WRKYGQK aminoacid sequence and a zinc-finger motif (C-X_4-7_-C-X_22-23_-H-X-H). A *cis*-acting DNA sequence motif C/TTGACC/T (termed W box), present in single or multiple copies in various promoters of plant genes, was demonstrated to bind WRKY TFs [8]. The WRKY TFs are encoded by a multigene family, with more than 70 and 100 members in Arabidopsis [9] and rice (*Oryza sativa*) [10], respectively. WRKY TFs are classified in different groups (I to III) on the basis of the number of WRKY domains and the features of the zinc-finger motif. Additional motifs present in some WRKY proteins are potential leucine zipper structures, known to allow protein dimerization [11,12]. The presence of the W box motif in several promoters of different members of the *WRKY* gene family, suggests a complex mechanism of autoregulation of their own expression [12]. Many reports have demonstrated that the transcription of *WRKY* genes is induced in plants after pathogen infection and, in turn, WRKY proteins may regulate the expression of several defense-related genes [9,13]. Along with their role in regulating the expression of several genes, information on the role of WRKY TFs in activating pathways of plant secondary metabolites also emerged including alkaloids, terpenoids, and phenylpropanoids [14,15] A *CjWRKY1* was identified in *Coptis japonica* and transiently expressed in *C. japonica* protoplasts inducing an increased expression of genes involved in the biosynthesis of the alkaloid berberine [16]. Xu et al. [17] identified a cotton *GaWRKY1* gene containing a leucine zipper motif and provided strong evidences on the role of this TF in the regulation of *CAD1-A* gene, involved in the biosynthesis of the sesquiterpene gossypol by means of interaction with the W-box present in the *CAD1-A* promoter. In *Catharanthus roseus* a CrWRKY positively regulated the terpenoid indole alkaloid biosynthesis [18]. OsWRKY89 and VvWRKY2 were identified to regulate the production of phenolic compounds such as lignin in rice and grape, respectively [19,20].

Because of the growing interest in bioactive metabolites of medicinal plants, together with the enhanced availability of genome sequencing information, the identification of transcription factors regulating specific secondary metabolism in these plants, may attract much attention. *Artemisia annua* L., belonging to the *Asteraceae* family, is known to be the plant source of artemisinin, a sesquiterpene lactone compound with effective antimalarial activity [21,22]. In previous works, we established suspension cell cultures of *Artemisia annua* able to produce artemisinin and further isoprenoid compounds; the significant enhancement of in vitro metabolite production, albeit with increases of different extent, was achieved by the application of methyl jasmonate (MeJa), miconazole or cyclodextrins (CD) [23,24,25]. Because of the high commercial value of artemisinin, an engineered microbial system was investigated and the semisynthetic production of artemisinin was reached in yeast [26]. Nevertheless, even today *Artemisia annua* plants are the main valuable source of artemisinin [27], thus elucidating the transcriptional regulatory networks of artemisinin biosynthesis may be very helpful to the goal of achieving highly producing plants. Ma et al. [28] described the isolation and characterization of a member of the WRKY transcription factor family (*AaWRKY1*), a member of group III, from the high artemisinin-yielding strain 001 *Artemisia annua*; AaWRKY1 protein binding to the W-box element was shown to activate the expression of artemisinin biosynthetic genes ADS, CYP71AV1, and DBR2 in leaves of *A. annua*. Recently, Chen et al. [7] identified a glandular trichome-specific WRKY transcription factor, AaGSW1, which positively regulated artemisinin biosynthesis. As reported by Xu et al. [17] a cotton *GaWRKY1* gene, belonging to the WRKY group IIa, was shown to be involved in the biosynthesis of the sesquiterpene gossypol. In order to explore if other members of *AaWRKY* family could be involved in the artemisinin metabolism we decided to investigate the *A. annua* WRKY gene homologous to *GaWRKY1*.

In the present work, we report on the isolation of a new WRKY (*AaWRKY40*) transcription factor in *Artemisia annua* homologous to the *Arabidopsis* group IIa *AtWRKY18/40/60* genes and to *GaWRKY1* of *Gossypium arboreum*. *AaWRKY40* encodes a leucine zipper-containing WRKY protein that localizes into the nucleus, and is induced in response to methyl jasmonate. Genome-wide analysis of *A. annua* WRKY transcription factors identified 122 *WRKY* genes, 8 of which belong to the group IIa that were further characterized. Protein motifs and regulatory sequence analysis of the WRKY of group IIa in the major Asteraceae species, and in the *AaWRKY40* closest homologs, identified a conserved bipartite W-box motif and a putative MeJa response element that may account for MeJa inducibility.

## 2. Results

### 2.1. Molecular Cloning of a Novel A. annua WRKY40 Gene

**Figure 1:**
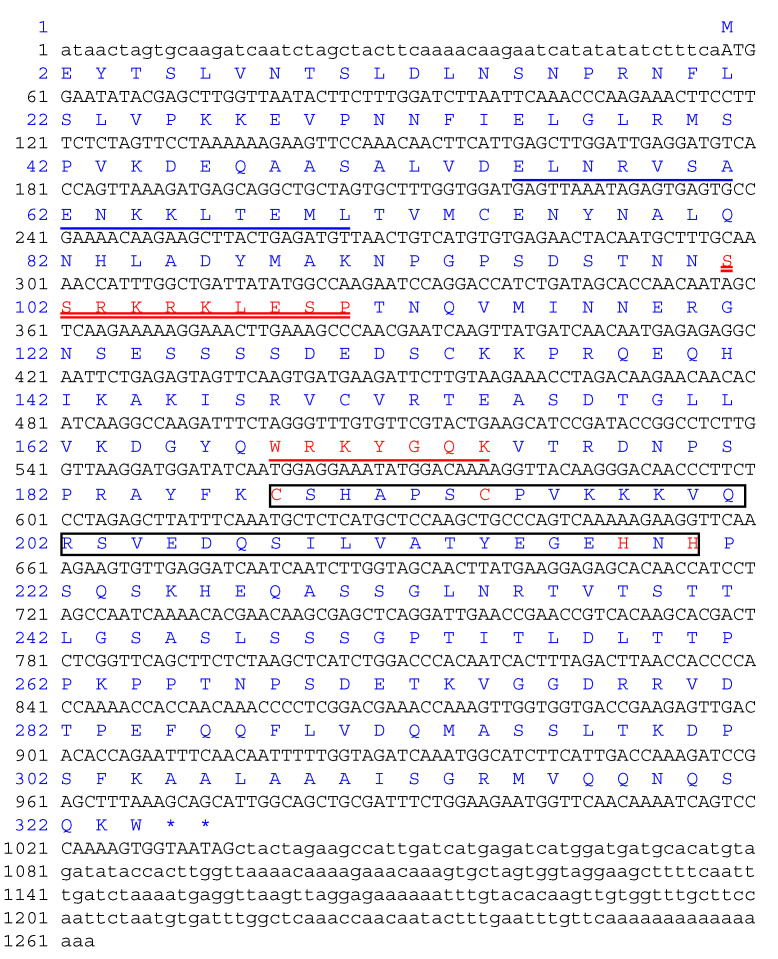
The nucleotide and deduced amino acid sequences of *AaWRKY40* cDNA. A WRKY domain containing an invariant WRKYGQK sequence is indicated in red and underlined, a putative zinc-finger motif is in **box** and the C2H2 motif in **red**. A nuclear localization signal (SSRKRKLESP) is marked in red and double underlined. Putative leucine zipper motif is underlined.

To identify the novel *A. annua* WRKY transcription factors possibly involved in artemisinin metabolism, an ortholog of *GaWRKY1* was isolated and characterized. Degenerate primers designed on the basis of the highly conserved domains of WRKY and leucine zipper of GaWRKY1 which was reported to influence the production of the sesquiterpene gossypol [17] were first used to amplify the 339-bp core fragment from *A. annua* cDNA as described in Materials and Methods. After sequencing and in silico translation of the DNA fragment, the Blast-X (https://blast.ncbi.nlm.nih.gov) program revealed a significant amino acid sequence similarity with other WRKY members reported in databases. In order to isolate the full-length coding sequence, genes-specific primers and the 3′ or 5′ universal primer of the RACE kit were used as described in Materials and Methods. This approach enabled us to obtain a full length ORF of a new *Artemisia annua* WRKY member submitted to the NCBI database (Accession number GU299481.1) here designated *AaWRKY40*. The full-length cDNA sequence of *AaWRKY40* consisted of 1263 nucleotides containing a 57-bp 5′ UTR, a 978-bp ORF, and a 228-bp 3′-UTR (Figure 1). *AaWRKY40* encoded a polypeptide containing 324 amino acid residues with a predicted theoretical molecular mass of 35.8 kD and an isoelectric point of 8.83 as calculated using the ExPASy-Bioinformatics Resource Portal (https://www.expasy.org/).

**Figure 2:**
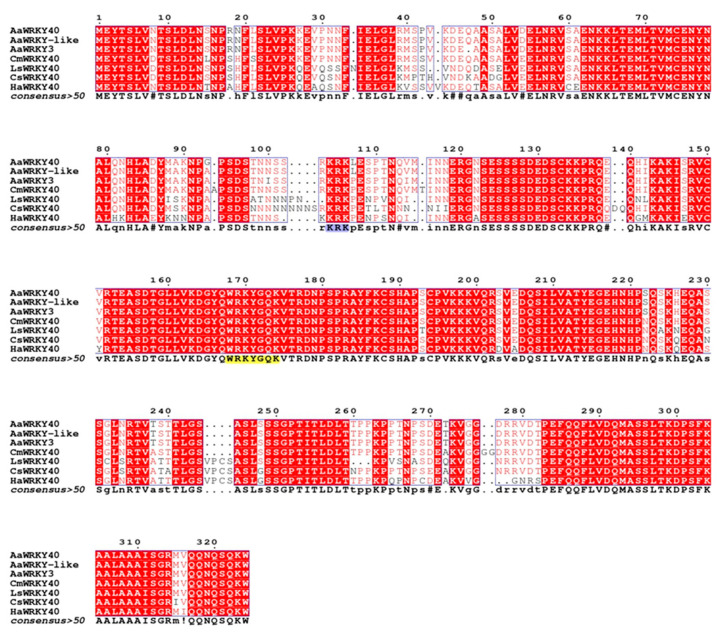
Alignment of AaWRK40 with related WRKY proteins from other Asteraceae plant species. The deduced amino acid sequence of AaWRK40 was aligned with Aa WRKY-like PWA73483.1, AaWRKY3 AGR40499.1, Cxm WRKY40 AJF11723.1, Ls WRKY40 XP_023744098.1, Ha WRKY40 XP_022015554.1(83%), CsWRKY40 XP_024969930.1, using MultAlign software [29] with default parameters. Predicted domains are indicated above the sequences.

To elucidate the genomic organization of *AaWRKY*40, specific primers (AaWRKY40-FullF/AaWRKY40-FullR) selected in the 5′ and 3′ regions of the *AaWRKY*40 cDNA sequence were used to amplify the corresponding genomic region. Comparing the nucleotide sequence of the 1739 bp genomic fragment with the cDNA coding sequence, *A. annua WRKY40* gene comprises four introns and five exons (Figure S1). The sequence analysis showed that the AaWRKY40 protein included one typical WRKY domain that contained a highly conserved amino acid sequence WRKYGQK and one putative (C2H2)-type zinc finger motif (C-X5-C-X23-H-X1-H) (Figure 1), suggesting that AaWRKY40 belongs to group II of the WRKY family [1]. In addition, AaWRKY40 has a putative leucine zipper in its N-terminal end (Figure 1). The deduced amino acid sequence of AaWRKY40 was analyzed using the BLAST-P program (https://blast.ncbi.nlm.nih.gov) and the results revealed that the AaWRKY polypeptide shared high degrees of identity with other plant species belonging to the *Asteraceae* family (Figure 2). The highest identity was observed toward *A. annua* WRKY-like, Sequence ID: PWA73483.1 (99%); *A. annua* WRKY, Sequence ID: AGR40499.1 (98%); *Chrysanthemum morifolium* WRKY40, Sequence ID: AJF11723.1 (95%); *Lactuca sativa* WRKY40, Sequence ID: XP_023744098.1 (85%); *Helianthus annuus* WRKY40, Sequence ID: XP_022015554.1 (83%); *Cynara cardunculus* var. *scolymus*. WRKY40, Sequence ID: XP_024969930.1 (84%).

### 2.2. AaWRKY40 Nuclear Localization in A. annua Protoplasts

**Figure 3:**
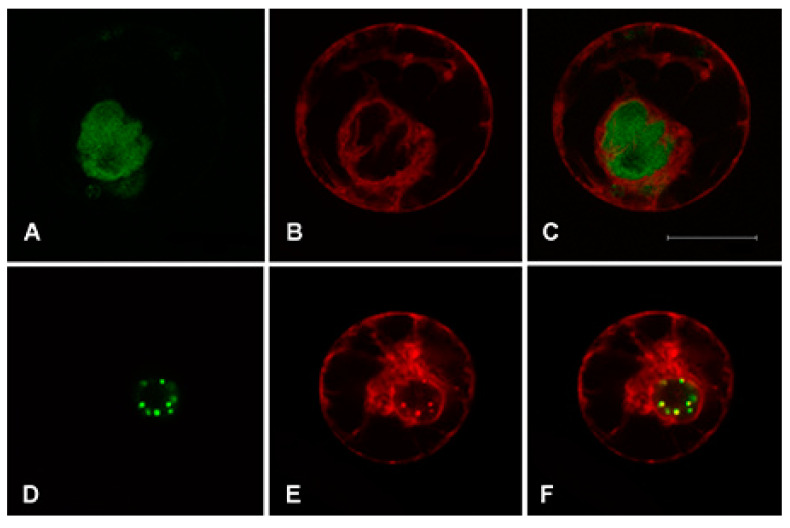
*Artemisia annua* protoplasts transiently expressing WRKY40:GFP and RFP-KDEL. Panels (**A**,**D**) show GFP signal in green; panels (**B**,**E**) show RFP signal in red and panels (**C**,**F**) show the merge of the two fluorescent signals producing yellow color if overlapping. Scale bar: 20 μm.

In order to investigate the intracellular localization of *AaWRKY40* in *A. annua* protoplasts, we carried out transient transformation experiments with the chimeric construct *WRKY40:GFP* observing a good nuclear localization compared with cytosolic GFP (Nisi R., PhD thesis, http://hdl.handle.net/1889/1332). To confirm the nuclear localization we combined the expression of construct *WRKY40:GFP* with the ER localized construct RFP-KDEL [30]. Protoplasts transformed with the two constructs clearly showed WRKY40:GFP green fluorescence (Figure 3A) perfectly delimited by the ER labelled by the RFP-KDEL red fluorescence (Figure 3B,C). In addition, we occasionally observed aggregation of GFP fluorescence within the nucleus indicating a possible localization in both the nucleoplasm and the nuclear bodies (Figure 3D–F). This localization was similar to that observed for the Arabidopsis WRKY40 and WRKY18 proteins [31].

### 2.3. Expression Analysis in A. annua Suspension Cell Cultures

**Figure 4:**
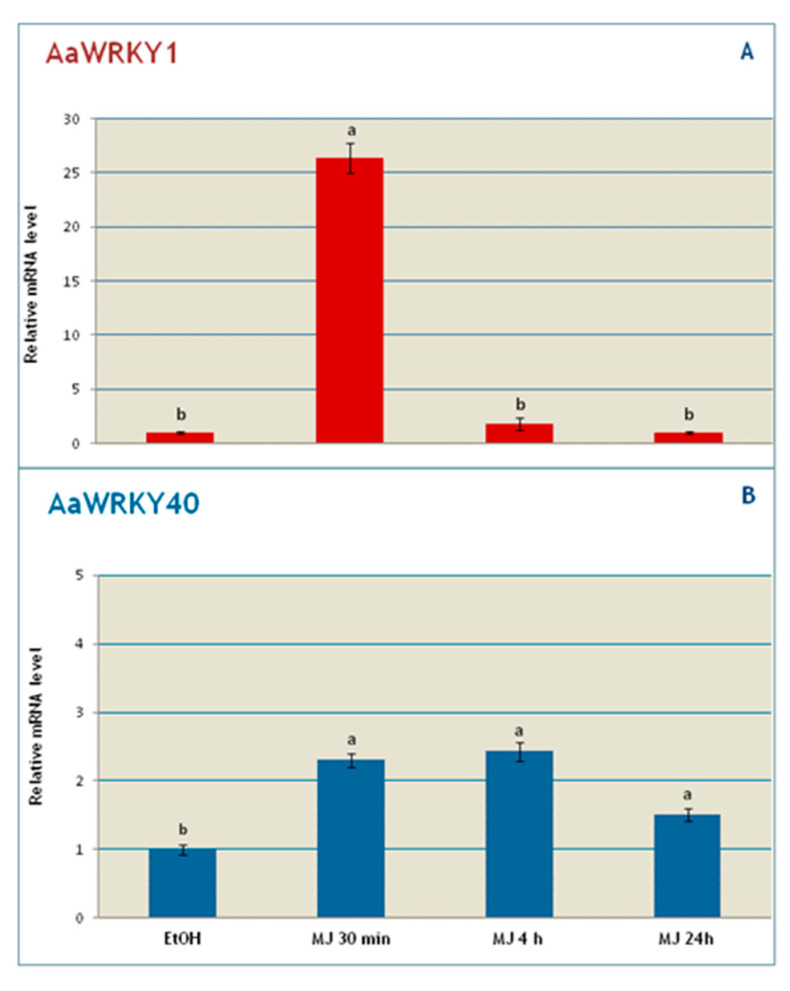
Estimation of the relative mRNA levels of *AaWRKY1* (**A**) and *AaWRKY40* (**B**) in A. annua suspension cultures treated with 22 µM methyl jasmonate (MJ). Ethanol (EtOH) was used to dissolve methyl jasmonate and added to control samples. Data were submitted to one-way ANOVA with Tukey’s post-hoc test, different letters denote significant differences between control and treatment (*p* < 0.05).

Quantitative expression analysis of *AaWRKY1* [28] and *AaWRKY40* genes in *A. annua* suspension cell cultures treated with 22 µM methyl jasmonate (MeJa) was evaluated at different time intervals. The results obtained showed a high induction of the expression of *AaWRKY1* soon after 30 min MeJA treatment up to more than 25-fold compared to the control (ethanol treated samples). After 4 h, *AaWRKY1* expression level dropped to values only slightly higher (1.78 fold) than the control and returned to steady state expression at 24 h (Figure 4A). *AaWRKY40* was also induced after 30 min MeJa treatment, although to a lower extent, with a 2.5-fold increase of gene expression. Differently from *AaWRKY1*, the expression of *AaWRKY40* maintained the 2.5-fold induction at 4 h and was still 1.5-fold higher than the control after 24 h (Figure 4B), highlighting a different kinetics of induction in response to MeJa.

### 2.4. Genome-Wide Identification of WRKY Transcription Factors in A. annua

**Figure 5:**
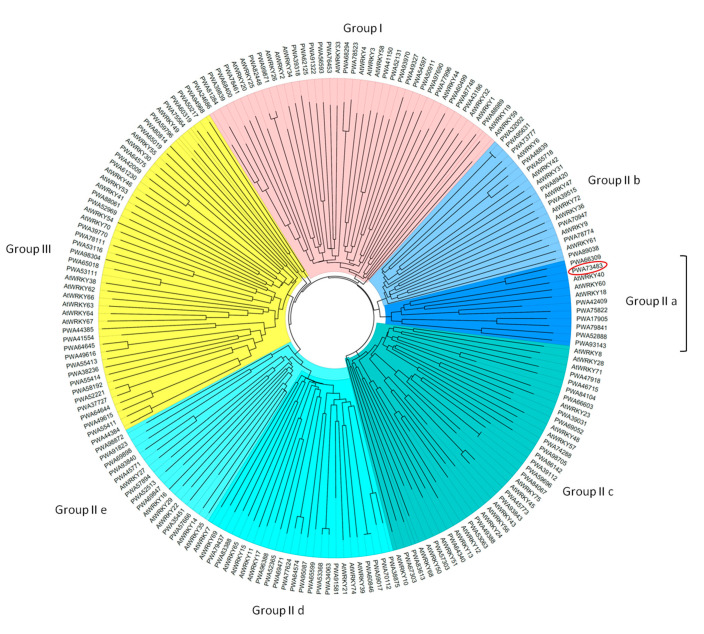
Phylogenetic tree of WRKY proteins from *A. annua* and *A. thaliana*. The complete amino acid sequences of 122 *A. annua* and 71 Arabidopsis WRKY proteins were aligned, and the phylogenetic tree constructed with 1000 bootstrap replicates, using Muscle EMBL-EBI analysis tool [35] followed by visualization with FigTree (http://tree.bio.ed.ac.uk/software/figtree/). WRKY groups and subgroups are highlighted with different colors: pink for group I, gradation of blue for group II, yellow for group III. The protein corresponding to the cloned *AaWRKY40* gene is circled in red.

The WRKYs are one of the largest families of plant transcription factors. The number of WRKY TFs varies in different species of higher plants, being 71 in Arabidopsis [9] and 174 in soybean [32]. With the completion of genome sequencing, the number of WRKY transcription factors in many species has been revealed. Recently, a high-quality draft genome sequence of *A. annua* was obtained and published [33]. We downloaded the genome, transcript, and peptide sequence data from the *A. annua* genome assembly (PRJNA416223) and searched for WRKY transcription factors. In total, 154 proteins annotated as WRKY transcription factors were extracted from the database. The genomic loci of the corresponding genes were checked, and transcripts matching the same genomic locus were trimmed to exclude annotation bias. After this filtering step, the proteins from the 145 genes corresponding to unique genomic loci were scanned against the PROSITE collection of motifs (https://prosite.expasy.org/scanprosite/). Of these, only 122 proteins showed domains indicating their belonging to the WRKY class of transcription factors. Among these, two presented high homology with WRKY proteins but were short and the WRKY domain was missing, likely because of partial peptide annotation. An *A. annua* database containing ID, annotations, transcript, and protein sequences of the 122 identified WRKY transcription factors was obtained (Table S1, *Artemisia annua* WRKY database). The whole protein sequences of the 122 *A. annua* WRKYs were compared with WRKY proteins from *Arabidopsis thaliana* and used to construct the phylogenetic tree shown in Figure 5. This analysis allowed to subdivide the WRKY transcription factors (TF) into the three major groups, and relative subgroups, previously identified in other plant species. Those possessing two heptapeptides are clustered into group I; both group I and II members harbor one C2H2 type zinc finger motif, while the group III members feature a C2HC one. The large size of group II has been addressed by its division into five subgroups (IIa, IIb, IIc, IId, and IIe), based on peptide sequence [11,34]. *AaWRKY40* gene corresponded to that coding for the *A. annua* annotated protein PWA73483, and fell into the group IIa comprising eight *A. annua* proteins homologous to *A. thaliana* WRKY 18, 40, 60. AaWRKY40/AaPWA73483 and AaPWA66309 were extremely similar (99.69% identity) but were encoded by genes from different genomic loci, indicating a recent duplication.

### 2.5. The Group IIa of WRKY Transcription Factors in A. annua

#### 2.5.1. Phylogenetic Analysis of WRKY Group IIa of *A. annua*

**Figure 6:**
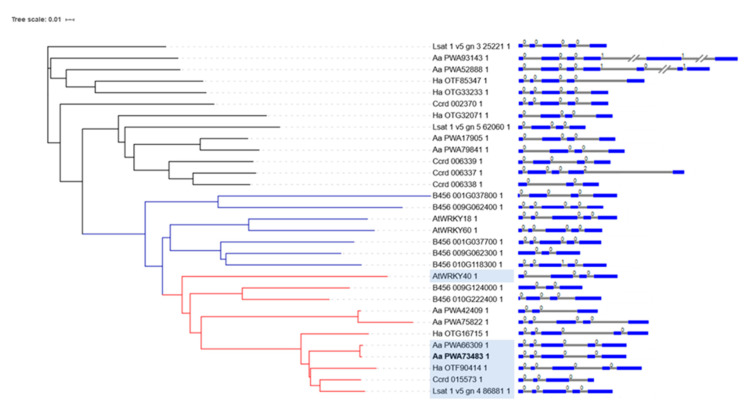
Phylogenetic tree of WRKY proteins from *A. annua* (Aa), *L. sativa* (Lsat), *C. cardunculus* (Ccrd), *H. annuus* (Ha), *A. thaliana* (At), and *G. raimondii* (B456). The complete amino acid sequences of the group IIa WRKY proteins were aligned, and the phylogenetic tree constructed with 1000 bootstrap replicates, using Muscle EMBL-EBI analysis tool [35] followed by visualization with iTOL [36]. The clades containing the WRKY proteins that are more closely related to the well characterized Arabidopsis WRKY18/60, or WRKY40 are highlighted in blue or red, respectively. Genomic exon-intron structure of the genes, shown on the right side, was visualized with PIECE 2.0 [37] exons are in blue, introns in grey.

To further characterize *AaWRKY40* and the other WRKY transcription factors belonging to the group IIa, available proteins, transcripts, genomic sequences and 3000 bp upstream promoter regulatory sequences from *A. annua,* three other Asteraceae reference species (*L. sativa*, *C. cardunculus*, *H. annuus*), *A. thaliana* and *Gossypium raimondii* as the reference sequenced genome for cotton, were retrieved (Table S2) and analyzed for a total of 31 genes/proteins. A phylogenetic tree of the corresponding WRKY proteins was obtained and is shown in Figure 6. The protein encoded by the *AaWRKY40* gene (*Aa PWA73483*) and the closest Aa PWA66309 protein, were highly related to AtWRKY40, and formed a subgroup of 11 proteins (Figure 6 in red) with high sequence identity. However, this group was enclosed in a wider group of highly similar proteins which also included AtWRKY18/60 and their homologues (Figure 6 in blue), known to be functionally correlated to AtWRKY40. The genomic intron-exon structure of these genes was also analyzed and is shown in Figure 6. Most of the group IIa genes displayed 3–4 introns and 4–5 exons, with the exception of four genes (two from *G. raimondii*, one from *A. annua* and one from *C. cardunculus*) displaying two introns, and two *A. annua* genes showing 5 and 6 introns.

#### 2.5.2. Conserved Protein Motifs in WRKY Group IIa of *A. annua*

**Figure 7:**
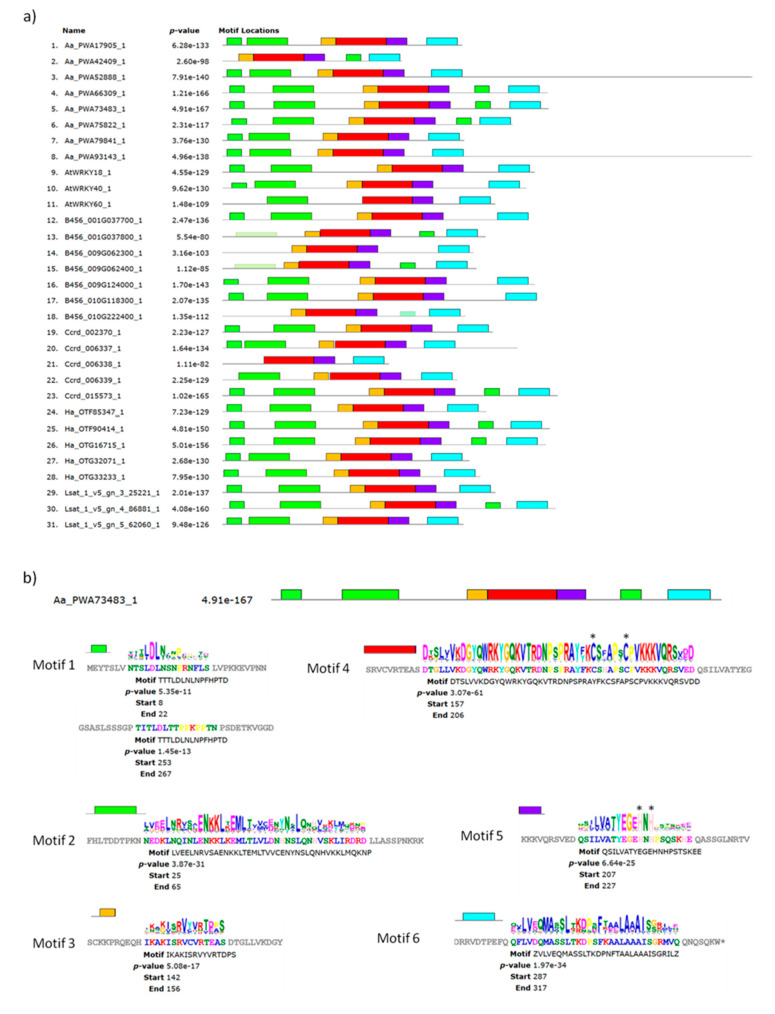
Results of the MEME analysis run on the 31 group IIa WRKY proteins from *A. annua* (Aa), *L. sativa* (Lsat), *C. cardunculus* (Ccrd), *H. annuus* (Ha), *A. thaliana* (At) and *G. raimondii* (B456), visualization of the six identified conserved domains with different colors (Motif 1, green; Motif 2, light green; Motif 3, orange; Motif 4, red; Motif 5, indigo; Motif 6, light blue) (**a**). Motifs and consensus sequences found in the AaWRKY40 (Aa_PWA73483_1) protein (**b**). Asterisks mark the invariant cysteines and histidines that are required to form the zinc-finger motif located in Motif 4 and 5.

Group IIa proteins were analyzed with MEME (Multiple Em for Motif Elicitation) (http://meme-suite.org) to identify the domains that were highly conserved. Among the six conserved motifs found, the one with the highest score corresponded to the WRKY core domain (Motif 4, red in Figure 7). The WRKY functional domain was indeed splat into three MEME motifs close to each other, Motif 3, 4, and 5 (orange, red and indigo in Figure 7a), with the invariant cysteines and histidines, required to form the zinc-finger motif, located in Motif 4 and 5, respectively (marked with asterisks in Figure 7b).

Motif 2 represented the leucine zipper at the N-terminus (light green in Figure 7) that characterizes the group IIa proteins and is involved in AtWRKY 18, 40, 60 protein–protein interactions. This leucin zipper motif is poorly or not conserved in four members of the cotton WRKY (protein number 13, 14, 15, and 18 in Figure 7a). Motif 1 (green in Figure 7) is a short motif at the N-terminus that precedes the leucin zipper and it is also absent from the same members of cotton WRKYs. However, these proteins may have an incomplete N-terminus. Interestingly, this motif is duplicated at the C-terminus of only few group IIa WRKY members, including AaWRKY40/AaPWA73483 and AaPWA66309, and identifies a subgroup of Asteraceae WRKY proteins likely evolved from the same ancestral gene. Motif 6 was highly conserved in all the group II proteins and contained invariant residues of leucine and adenine of unknown function. Also, a strong nuclear localization motif was found between position 90 and 100 aa in the AaWRKY40/AaPWA73483 protein using NucPred (https://nucpred.bioinfo.se/cgi-bin/multi.cgi), that confirmed the observed nuclear localization of the GFP fusion protein in protoplasts transformation assays. The exon–intron structure of the 31 group IIa genes was also analyzed and it is shown in Figure 6. The number of exons varied from 3 to 9, with most genes (25 out of 31) containing 4–5 exons.

### 2.6. Regulatory Motifs in the Upstream Regions of Group IIa WRKY Transcription Factors

The 3000 bp upstream nucleotide sequences of group IIa WRKY genes (Table S3) were searched for regulatory motifs that included W-box (C/TTGACT/C) and various jasmonate-responsive elements (G-box, CGTCA motifs, and GCC box). Several CGTCA motifs involved in MeJa responsiveness and W-box elements were generally present in the group IIa of WRKY TFs (Table 1 and Table S4), with some genes particularly enriched in such regulatory elements. These genes with high score included *AaWRKY40/AaPWA73483* and *AaPWA66309*, that displayed five and seven MeJa-RE, respectively, and five W-box.

**Figure 8:**
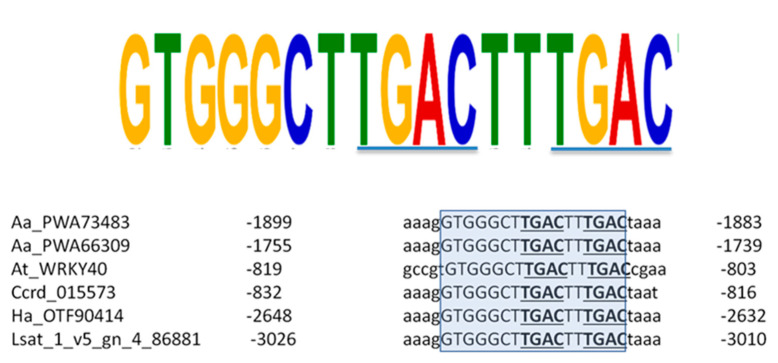
Results of the MEME analysis run on the promoter of WRKYs in group IIa. The highly conserved 17 nucleotides long sequence is shown. Conserved W-box TGAC core sequences are underlined. The identified double W-box is highlighted in light blue in the sequence alignment. Aa: *Artemisia annua*; At: *Arabidopsis thaliana*; Ccrd: *Cynara cardunculus*; Ha: *Helianthus annus*; Lsat: *Lactuca sativa*.

The 3000 bp promoter regions of the group IIa WRKY were further analyzed with MEME (Multiple Em for Motif Elicitation). The analysis identified a long stretch of conserved nucleotide residues that contained two adjacent W-box DNA binding motifs (Figure 8). This motif was present in six genes: five encoding the highly similar proteins that form a specific subclade containing AaWRKY40/AaPWA73483 and AaPWA66309 in the phylogenetic tree of group IIa WRKYs (Figure 6), and AtWRKY40.

In the conserved motif, the two W-box core sequences TGAC were spaced by two nucleotides (TTGACTTTGACT/C). This double W-box was placed more than 2000 bp upstream of the ATG in *A. annua*, *H. annuus,* and *L. sativa*, whereas it was placed at around −800 bp in both *C. cardunculus* and *A. thaliana*. In the Asteraceae species, the conservation of the region harboring the double W-box motif was extended to 25 nucleotides, with a percentage identity ranging from 88 to 100%. These findings suggest that this element can be an important regulatory motif conserved between *AtWRKY40* and its homologues in Asteraceae species.

In *A. annua* at least three WRKY genes, *AaWRKY1* [28,38,39], *GLANDULAR TRICHOME-SPECIFIC WRKY 1 (AaGSW1)* [40], and *AA213240* [33] were shown or suggested to be involved in artemisinin production. We retrieved the sequences of these genes, that in our sequenced genome-based database corresponded to *AaPWA52969*, *AaPWA39112,* and *AaPWA78774*, and compared the promoters of these WRKY genes with those of *AaWRKY40/AaPWA73483* and *AaPWA66309.* All the three genes involved in the regulation of the artemisinin pathway were similarly enriched in W-box, G-box and MeJa-RE elements (Table 2), indicating that *AaWRKY40/AaPWA73483* and *AaPWA66309* genes might express in response to similar stimuli and co-regulated with the WRKYs that play a role in artemisinin production. In light of the high sequence homology between *AaWRKY40/AaPWA73483* and *AaPWA66309*, we cannot exclude that the reported expression data (Figure 4) represent the expression of both members.

## 3. Discussion

Plant growth and development are continually influenced by numerous environmental factors. Biotic and abiotic stresses affect the plant transcriptome, and the earliest defense responses consist of transcription regulation involving different TFs.

The WRKY family is among the main transcription factors that regulate plant responses to various stresses and phytohormones [41,42].

In the present work, a new WRKY TF (named AaWRKY40) of *Artemisia annua* has been identified to be orthologue of *AtWRKY40*. Its amino acidic sequence, tagged with a fluorescent protein, confirmed the nuclear localization expected for a transcription factor. Despite no interpretation of the phenomenon can be given at this stage, we also observed for our chimeric construct the possibility to form aggregates inside the nucleus as seen for other TFs, such as the nuclear-localized FBP9-YFP [43]. The use of protoplasts as experimental system is well established [44] and proved to be reliable and direct for tagged protein localization [45].

By analyzing AaWRKY40 amino acid sequence, a close similarity to WRKY40 of *C. morifolium*, WRKY40 of *L. sativa*, WRKY40 of *H. annuus*, WRKY40 of *C. scolymus* was detected; all these species, including *A. annua*, belong to the *Asteraceae* family. Furthermore, phylogenetic analysis showed that AaWRKY40 clustered with GaWRKY1 and AtWRKY40 in the group IIa. This close relationship of AaWRKY40 with TFs from other plant species, may indicate their probable common biological function. In cotton suspension cultured cells treated with a fungal elicitor and MeJa, the expression of both *GaWRKY1* and *CAD1-A* was induced, suggesting that GaWRKY1 and CAD1-A are components of the same pathway of cotton defense response, which involved the biosynthesis of the sesquiterpene gossypol [17]. In *Arabidopsis thaliana*, extensive functional analysis of different WRKY members identified a defined cluster in the group IIa constituted by WRKY18, WRKY40, and WRKY60, all of them MeJa-responsive [3]. The three WRKY proteins form either homocomplexes or heterocomplexes through leucine zipper motifs and functionally interact to affect plant defense responses. It is known that MeJa can act as an elicitor of secondary metabolism through the activation of various transcription factors [46]. Among these, members of the WRKY family were reported to be involved in the biosynthetic regulation of valuable plant metabolites, including terpenoids [15]. In hairy roots of *Salvia sclarea* the overexpression of *AtWRKY40* enhanced the levels of abietane diterpenes by activating the MEP-derived biosynthetic pathway [47].

In *Artemisia annua*, *AaWRKY1* [28] was identified and characterized to be involved in the regulation of artemisinin biosynthetic pathway. Specifically, its expression correlated with the expression of *ADS*, which is the first key enzyme in artemisinin biosynthetic pathway. Moreover, Jiang et al. [39] showed that transgenic *Artemisia annua* overexpressing *AaWRKY1* led to an enhanced content of artemisinin and the expression of *ADS* and *CYP*, the key enzymes in artemisinin biosynthetic pathway, was dramatically increased. Recently, Chen et al. [40] characterized a glandular trichome-specific *WRKY1 (GTSW1)* and showed that its overexpression in *A. annua* significantly improved artemisinin and dihydroartemisinic acid contents.

In this work, the same MeJa-elicitation conditions previously shown to significantly enhance artemisinin production in Artemisia cell cultures [23] resulted in the expected induction of *AaWRKY1* but also in the induction of *AaWRKY40* gene expression, with different kinetics that may be “linked” to their belonging to different phylogenetic groups. However, these similar expression profiles in response to MeJa-elicitation conditions that enhance artemisinin production may suggest an involvement of *AaWRKY40* in terpenoid metabolism. A possible mutual regulation through the W-box domains in the upstream regulatory sequence of these genes may potentially occur, and protein–protein interaction between class IIa and class III members of WRKY has been reported in *Arabidopsis* (Arabidopsis Interactome Mapping Consortium, 2011). Further research will help deepening the possible regulatory interactions between WRKY TFs in *A. annua*.

With the development of high-throughput sequencing technologies, the whole genome sequences of several plant species have been made publicly available. Large and variable numbers of WRKY TFs have been found in different plant species, likely reflecting their complex role in plant growth and response to abiotic and biotic stress [48]. Recently, WRKY transcription factors from sequenced genomes of six species of the superorder Asteranae were identified, and phylogenetic relationships, gene structures, cis-acting elements, and WRKY gene duplication events analyzed [49]. This analysis did not include *A. annua*, whose genome is also currently available [33]. By an extensive bioinformatic analysis of the whole genome sequences data (PRJNA416223) we identified 122 WRKY genes of *A. annua* that are encoded by different genome loci. This number differs from the estimation done by Shen et al. (137 members) [33] as we manually refined the annotation and excluded genes that were redundantly or wrongly annotated in the original assembly. Based on the whole amino acid sequence, the 122 AaWRKY were classified within the three main phylogenetic groups I, II, and III, with the second group further categorized into five subgroups (IIa–IIe) [34]. The WRKY family is significantly expanded in *A. annua* with respect to other Asteraceae species like *L. sativa*, *Daucus carota,* and *Panax ginseng* that have a number of WRKY genes comparable to Arabidopsis (ranging from 56 to 74) [49]. We focused our attention on the group IIa, to which the cloned *AaWRKY40* gene belongs. Group IIa harbors eight *A. annua* genes against the three genes found in *A. thaliana*. This amplification of class IIa WRKYs is not observed in all the Asteraceae species: *L. sativa* and *D. carota* genomes only contain three members of class IIa, whereas *C. cardunculus*, *P. ginseng* (Ginseng) and *H. annuus* contain five class IIa WRKYs. A gene very similar to *AaWRKY40*, coding for the Aa_PWA66309 protein, showed 99.49% nucleotide and 99.69% amino acid identity, and may derive from a recent duplication. The protein sequence structure and comparative analysis of AaWRKY40 showed that it has a single WRKYGQK conserved domain followed by a C2H2 type zinc-finger motif. Also, a putative leucine zipper motif was present toward the N terminus [50,51] as reported for other WRKY members such as ABF2 and WIZZ [52,53]. Function of the leucine zipper was proposed to mediate dimerization and increase the DNA-binding affinity of WRKY proteins [51]. Furthermore, a nuclear localization domain was identified in silico in agreement with the intracellular localization by means of GFP.

As reported in previous studies in other plant species the WRKYGQK domain can bind to W-box cis-elements with a C/TTGACC/T core sequence to activate downstream genes [11]. Also, it is well-known that the expression of different members of the WRKY family are influenced by the binding of the WRKYGQK domain to the W-box located upstream the coding sequence of other WRKY members. In our analysis of the upstream regulatory region of *AaWRKY40* gene, five W-box were identified. Particularly interesting was the presence of a double W-box (TTGACTTTGACT/C) that was highly conserved among the members of class IIa WRKY genes in the Asteraceae family, and this element was also present in the regulatory region of the Arabidopsis *WRKY40* (Figure 8). This conserved region may therefore represent a key regulatory element shared by WRKY40 orthologs, and its biological role should be further investigated. Besides W-box elements, one G-box, one GCC-box, two ERE and five to seven MeJa responsive elements were also found in the promoter regions of *AaWRKY40* and its close homolog, respectively. The presence of different types of MeJa responsive elements are consistent with the rapid induction of the *AaWRKY40* transcript observed in *A. annua* cell cultures in response to MeJa. According to the heat map of WRKY gene expression published in Shen et al. [33], *AaWRKY40* and its close homolog (identified in the paper with their locus ID AA259860 and AA329040, respectively), are widely expressed in all the plant tissues analyzed, and are abundant in leaves, stems, and flowers where artemisinin is produced. It would be worth conducting further functional analyses to assess whether the identified *A. annua* orthologs of WRKY40 could be involved in the secondary metabolism leading to artemisinin production. Several TFs regulating secondary metabolism are responsive to MeJa, and some WRKYs regulate the production of valuable natural products by modulating the transcription of the biosynthetic genes involved in the production of phenylpropanoids, alkaloids and terpenes (https://doi.org/10.1104/pp.114.251769). Recently, the Arabidopsis WRKY18 and WRKY40 were shown to activate the transcription of genes of the MEP-pathway to enhance the synthesis of bioactive abietane diterpenes in transgenic roots of *Salvia sclarea* [47]. In particular, overexpression of AtWRK40 was able to activate preferentially the transcription of a *deoxyxylulose 5-phosphate synthase* (*DXS*) gene, encoding the first committed step of the MEP-pathway and the *copalyl diphoshate synthase* (*CPPS*) gene, the precursor of several plant diterpenes.

The identification of the *A. annua* ortholog of AtWRKY40 and, more generally, the identification and precise annotation of WRKY transcription factors in a species of great interest for secondary metabolite production, can constitute a valuable tool to better define the complex regulatory pathways acting in *Artemisia annua* leading to the biosynthesis and accumulation of valuable metabolites such as the antimalaric artemisinin.

## 4. Materials and Methods

### 4.1. Plant Material and Chemical Treatments

*Artemisia annua* L. suspension cultures were established as previously described [23] and maintained in G6 medium: Murashige and Skoog medium (MS, [54] containing 2 mg·L^−1^ of 2.4D and 0.15 mg·L^−1^ BAP. The cultures were maintained on a rotary shaker (120 rpm) in continuous light conditions at 25 °C and were regularly subcultivated every 35 days by transferring 15 mL of 35 day-old suspensions into 85 mL fresh G6 medium. Chemical treatments were carried out on 15 day-old suspension cultures. The suspension cultures (25 mL) were incubated in the G6 medium containing 5 mg/L (22 µM) methyl jasmonate dissolved in ethanol 95% (Sigma, St. Luis, MO, USA) for 30 min, 4 h, 24 h, respectively. Equivalent volumes of ethanol were used as controls. After harvesting, the materials were immediately frozen in liquid nitrogen and stored at −80 °C for further analysis. *A. annua* protoplasts were isolated from suspension cultures and from in vitro cultured leaves. The preparation was identical, except for the need of cutting leaves, as described in the protocol of Maliga and co-workers [55].

### 4.2. Nucleic Acids Extraction and cDNA Preparation

*A. annua* leaves were frozen in liquid nitrogen and ground to a fine powder with a pestle in a pre-cooled mortar. Suspension cultures were filtered using nylon filters (30 µm pores size), frozen in liquid nitrogen, and powdered as for leaves and the powder was stored at −80 °C until used. Protoplasts were collected by centrifugation (5 min at 60× *g*) and frozen in liquid nitrogen. DNA was extracted using DNeasy Plant Mini Kit (Qiagen, Milan, Italy). RNA was isolated using SV Total RNA Isolation System (Promega, s.r.l., Milan, Italy). Nucleic acid concentrations were determined spectrophotometrically and the quality were checked by agarose gel electrophoresis. The first-strand cDNAs were obtained starting from 1 µg of total RNA using random hexamers and the ImProm-II Reverse Transcription System (Promega) according to Manufacturer’s instructions.

### 4.3. Cloning and Sequence Analysis

In order to obtain internal conserved fragments, degenerate primers AaLeuDegF/AaWRKYDegR (Table 3) were designed based on the reported GaWRKY1 protein sequence [17]. Then, the conserved sequence of AaWRKY was amplified using the polymerase chain reaction (PCR). Subsequently, based on the sequence information of the DNA fragment obtained, the 3′ and 5′region were identified using specific primers and rapid amplification of cDNA ends using the 3′ RACE System (Life Technologies Ltd., Paisley, UK). RACE1F/ RACE1R were used for the isolation of the 3′ region and RACE2F/RACE2R were used for the 5′ region of AaWRKY40 according to the manufacturer’s instructions. Finally, a pair of gene-specific primers (AaWRKY40-FullF/AaWRKY40-FullR) was designed and used to amplify the full-length sequence of AaWRKY40 and the corresponding genomic region. The amplified products were then cloned into pGEM-T Easy vector (Promega Italia s.r.l., Milano) and used to transform competent *E. coli* strain XL1-Blue (Stratagene) using standard procedures. Recombinant DNA plasmid were extracted using Qiagen plasmid midi Kit (Qiagen, Milano, Italia) and the nucleotide sequence were performed using an ABI 3130 DNA Sequencer (Applied Biosystems). The primers used in this study are listed in Table 3.

### 4.4. Preparation of Gene Constructs and Protoplast Transformation

*WRKY40:GFP* construct was obtained by inserting the AaWRKY40 cDNA in a pGY1 derived vector containing green fluorescent protein (GFP), namely Aleu-GFP [56] as a BamHI/NheI fragment. Restriction sites were inserted by amplifying the AaWRKY40 cDNA from recombinant plasmid with BamFor (ggatccTTCAATGGAATATACCAGCTTGGT) and NheRev (gctagcCCACTTTTGGGACTGATTTTGTTGA) primers. *A. annua* protoplasts were transformed using PEG direct gene transfer performed essentially as described by Di Sansebastiano and co-workers [57]. The new WRKY:GFP construct was compared with the cytosolic construct GFP and used in combination with the ER localized construct RFP-KDEL [30]. About twenty to thirty micrograms of plasmid DNA were used for the transformation of about 400,000 protoplasts. Transformation efficiency depends on the amount of supercoiled plasmid DNA so it can vary independently from the measurable quantity of DNA. In the case of the non-GFP-tagged gene (WRKY), where no visual screening was possible, 30 micrograms DNA were used to guarantee overloading. The transformation efficiency of the reported experiments was around 50%. All visual analyses were performed 18–24 h after transformation.

Protoplasts transiently expressing fluorescent constructs were observed by fluorescence microscopy in their culture medium. They were examined with a confocal laser-microscope LSM 710 Zeiss (ZEN Software, GmbH, Germany) mounting material in water [58]. GFP was detected within the short 505–530 nm wavelength range, assigning the green color, RFP within 560–615 nm assigning the red color. Excitation wavelengths of 488 and 543 nm were used. The laser power was set to a minimum and appropriate controls were made to ensure there was no bleed-through from one channel to the other. Distribution of fluorescent protein was observed in three independent replicates from temporally independent experiments. Images were processed using Adobe Photoshop 7.0 software (Mountain View, CA, USA).

### 4.5. Expression Analysis of AaWRKY

Quantitative real time PCR experiments were performed using an Applied Biosystem 7500 apparatus using ubiquitin (UBQ), accession number (EU258763) as housekeeping gene. The probes were labelled at 5′ with 6-carboxy-fluorescein (FAM) and at 3′ with Tetramethylrhodamine (TAMRA). The primers and probes used for *AaWRKY1* and *AaWRKY40* are listed in Table 3 and were purchased from PRIMM srl (Milan, Italy). Amplifications were performed in a total volume of 25 µL mix containing the sequence-specific primer set (900 nM each primer), the specific probe (200 nM), 0.5 µL of the first strand cDNA, and 12.5 µL of 2X TaqMan Universal PCR Master Mix (Applied Biosystems, Foster City, CA, USA). Experiments were conducted in triplicates using cDNAs obtained from two independent experiments. Real-time PCR cycles were as follows: 50 °C, 2 min (1 hold); 95 °C 10 min (1 hold); 95 °C, 15 s, 60 °C, 1 min (40 cycles). Quantification of transcripts was carried out using comparative quantitation module based on the 2^-ΔΔ*C*^_T_ method [59]. The relative expression was normalized against ubiquitin, and calculated using the untreated samples as a calibrator, whose expression was given equal to one.

### 4.6. Identification of WRKY Genes in the Genome of A. annua

The genome, transcript and peptide sequence data from the genome assembly of *A. annua* [33] were downloaded from NCBI (PRJNA416223). Proteins annotated for WRKY domain were extracted (a total of 164) and corresponding transcripts were searched in the *A. annua* genome with a local BLAST program (https://blast.ncbi.nlm.nih.gov) to trimmed genes for unique genomic loci. True homology to WRKY proteins was assessed using PROSITE (https://prosite.expasy.org/) to score the domains that characterize WRKY proteins. Out of 164 annotated WRKY, only 122 proteins fulfilled our criteria and were included in the WRKY database (Table S1, *Artemisia annua* WRKY database).

### 4.7. Phylogeny, Protein Conserved Motif and Gene Structure Analysis of AaWRKY Genes

The whole protein sequences of the identified 122 AaWRKY were aligned and compared with those of *A. thaliana* to construct a neighbor-joining phylogenetic tree constructed with 1000 bootstrap replicates, using Muscle EMBL-EBI analysis tool [35]. The phylogenetic tree was then visualized with FigTree (http://tree.bio.ed.ac.uk/software/figtree/) and WRKY proteins were divided into groups based on domain characteristics and phylogenetic relationships. The alignment and phylogenetic tree of the 31 group IIa WRKY proteins from *A. annua*, *L. sativa*, *C. cardunculus*, *H. annuus*, *A. thaliana* and *G. raimondii*, was also carried out using Muscle EMBL-EBI analysis tool followed by visualization with iTOL [36]. WRKY protein conserved motifs were analyzed in MEME (http://meme-suite.org) with the following parameters: maximum number of motifs = 6, and motif length = 6–50 residues. Genomic exon-intron structure of the genes was visualized with PIECE 2.0 [37]). Nuclear localization signals were predicted with NucPred (https://nucpred.bioinfo.se/cgi-bin/multi.cgi).

### 4.8. Cis-Acting Element Analysis

To identify cis-acting elements of group IIa WRKY genes, sequences of 3000 bp length located upstream of the ATG were retrieved from the genomes of *A. annua*, *L. sativa*, *C. cardunculus*, *H. annuus*, *A. thaliana* and *G. raimondii,* either using Ensembl Plants and TAIR tools or NCBI (Table S2). The promoter of *AtWRKY40* was first analyzed using PLANTCARE (http://bioinformatics.psb.ugent.be/webtools/plantcare/html/). The promoters for the 31 group IIa WRKY genes were then searched for specific motifs using RSAT Plants [60]. The motifs searched were the following: the WRKY W-box binding site (C/TTGACT/C); jasmonate response (MeJa-RE, CGTCA) and defense-related motifs (GCC-box: GCCGCC; G-box: CACGTG); ethylene-responsive element (ERE: ATTTCAAA, ATTTCATA); cis-acting element involved in salicylic acid responsiveness (TCA-element: CCATCTTTTT, TCAGAAGAGG).

### 4.9. Statistical Analysis

The data are presented as the mean value of three independent replicate experiments (n = 3) with standard deviation. One-way analysis of variance (ANOVA) with Tukey’s post-hoc test was applied to establish significant differences between control and treatment (*p* < 0.05). All statistical comparisons were performed using SigmaStat version 11.0 software (Systat Software Inc., Chicago, IL, USA).

## Figures and Tables

**Table 1:** RSAT analysis of the cis-regulatory elements in the promoters of group IIa WRKY genes.

seq	W-Box	G-Box	GCC-Box	TCA-Element	MeJa-RE	ERE
**Aa_PWA66309**	**5**	1	1	0	**7**	2
**Aa_WRKY40**	**5**	1	1	0	**5**	2
**At_WRKY18**	**6**	0	1	0	**5**	0
**At_WRKY40**	**5**	0	0	0	**5**	1
B456_001G037700	6	1	0	0	5	8
Ccrd_002370	6	1	0	0	5	2
Lsat_1_v5_gn_3_25221	7	0	0	0	5	0
Aa_PWA93143	4	0	0	0	4	1
B456_001G037800	7	1	1	0	4	1
Ha_OTF90414	4	0	0	0	4	3
Lsat_1_v5_gn_5_62060	6	0	0	0	4	2
Aa_PWA52888	4	0	0	0	3	1
Aa_PWA75822	3	0	0	0	3	2
B456_009G062400	9	0	0	0	3	1
Ccrd_006339	4	0	0	0	3	3
Ha_OTF85347	4	0	0	0	3	7
Ha_OTG16715	6	0	0	0	3	1
Ha_OTG32071	2	0	3	0	3	4
Ha_OTG33233	4	2	0	0	3	3
Aa_PWA17905	6	0	0	0	2	0
Lsat_1_v5_gn_4_86881	10	1	0	0	2	4
Aa_PWA79841	7	0	0	0	1	1
At_WRKY60	8	0	0	0	1	1
B456_009G062300	6	1	0	0	1	4
B456_010G118300	6	0	0	0	1	10
B456_010G222400	6	0	0	1	1	3
Ccrd_006337	2	0	0	0	1	2
Ccrd_006338	2	0	0	0	1	2
Aa_PWA42409	3	0	0	0	0	0
B456_009G124000	7	0	0	0	0	8
Ccrd_015573	6	1	0	0	0	2
**total**	**166**	**10**	**7**	**1**	**88**	**81**

**Table 2:** RSAT analysis of the cis-regulatory elements in the promoters of selected WRKY genes.

seq	W-Box	G-Box	GCC-Box	TCA-Element	MeJa-RE	ERE
AaGSW1/Aa_PWA39112	10	1	2	0	4	0
AaWRKY1/Aa_PWA52969	8	1	0	0	4	2
AA2132240/Aa_PWA78774	7	0	1	0	3	0
Aa_PWA73483	5	1	1	0	5	2
Aa_PWA66309	5	1	1	0	7	2
**total**	35	4	5	0	23	6

**Table 3:** Primer nucleotide sequences.

Primer	Sequence	Application
AaWRKYDegR	YTTYTGICCRTAYTTICKCCA	RT-PCR
AaLeuDegF	GARAAYAARAARYTIACIGAR	RT-PCR
3′ GSP1-F	TGGCCAAGAATCCAGGAC	3′RACE
3′ GSP2-F	GAGAGAGGCAATTCTGAG	3′RACE
5′GSP1-R	GTCCTGGATTCTTGGCCA	5′RACE
5′GSP2-R	CTCAGAATTGCCTCTCTC	5′RACE
AaWRKY40-Full-F	CTAGTGCAAGATCAATCTAG	RT-PCR/Genomic
AaWRKY40-Full-R	CATGATCTCATGATCAATGG	RT-PCR/Genomic
RTWRKY40for	GGACAACCCTTCTCCTAGAGCTT	qRT-PCR
RTWRKY40rev	GCTCTCCTTCATAAGTTGCTACCAA	qRT-PCR
RTWRKY40probe	AATGCTCTCATGCTCCAAGCTGCCC	qRT-PCR
RTWRKY1for	GGAAACACACTTGCAACCATCA	qRT-PCR
RTWRKY1rev	GTGGTGGGTTGTGTTTATTTCATG	qRT-PCR
RTWRKY1probe	CTCGTTTGGCCGAACCACCTTTGC	qRT-PCR
RTUBIfor	CGGACCAGCAGAGGTTGATATT	qRT-PCR
RTUBIrev	CAGCCTTAAGACCAAATGGAGAGT	qRT-PCR
RTUBIprobe	CAGGAAAGCAGCTTGAAGATGGCCG	qRT-PCR

## Supplementary Materials

The following are available online at https://www.mdpi.com/2223-7747/9/12/1669/s1. Figure S1: Nucleotide and deduced amino acid sequences of *AaWRKY40* genomic DNA. Table S1: AaWRKY_database. Table S2: MEME analysis of protein sequences. Table S3: promoter sequences. Table S4: results of RSAT analyses.
