# Supplementary material for: Genome-Wide Identification of WRKY Genes in Artemisia annua: Characterization of a Putative Ortholog of AtWRKY40
Source: Plants (Basel). 2020 Nov 28;9(12):1669. doi: 10.3390/plants9121669 (PMC7761028; doi:10.3390/plants9121669)
Supplement: Supplementary file 1 [file plants-09-01669-s001.zip › Suppl_rev/Fig S1.pdf]

```
   1                                                      M  E  Y  T  S  L  V  N
   1 ctagtgcaagatcaatctagctacttcaaaacaagaatcatatatatctttcaATGGAATATACCAGCTTGGTTA
   9   T  S  L  D  L  N  S  N  P  R  N  F  L  S  L  V  P
  76 ATACTTCTTTGGATCTTAATTCAAACCCAAGAAACTTCCTTTCTCTGGTTCCTgtaagttcttaaacatatacat
 151 acatcatatatatgaacatacatatatttacatgtgatggactttagctaacaaggttccttgatttttgacttg
  26    K  K  E  V  P  N  N  F  I  E  L  G  L  R  M  S  P  V  K  D  E
 226 tagAAAAAAGAAGTTCCAAACAACTTCATTGAGCTTGGGTTGAGGATGTCACCAGTTAAAGATGAGgtatacatc
 301 atttcacataatccatgatataaatgctttctgtctaaaattacatatgtttagctatagctatatgaataacat
  47                             Q  A  A  S  A  L  V  D  E  L  N  R  V  S  A  E
 376 attcgggttttgtgggaatttttaatagCAGGCTGCAAGTGCTTTGGTGGATGAGTTAAATAGAGTGAGTGCTGA
  63  N  K  K  L  T  E  M  L  T  V  M  C  E  T  Y  N  A  L  Q  N  H  L  A  D  Y
 451 AAACAAGAAGCTTACTGAGATGTTAACTGTCATGTGTGAGACCTACAATGCTTTGCAAAACCATTTGGCTGATTA
  88  M  A  K  N  P  G  P  S  D  S  T  N  N  S  S  R  K  R  K  L  E  S  P  T  N
 526 TATGGCTAAGAATCCAGGACCATCTGATAGCACCAACAATAGTTCAAGAAAAAGGAAACTTGAAAGCCCAACGAA
 113  Q  V  M  I  N  N  E  R  G  N  S  E  S  S  P  S  D  E  G  S  C  K  K  P  R
 601 TCAAGTTATGATCAACAATGAGAGAGGCAATTCTGAGAGTAGTCCAAGTGATGAAGGTTCTTGTAAGAAACCTAG
 138  Q  E  Q  H  I  K  A  K  I  S  R  V  C  V  R  T  E  A  S  D  T  G  L
 676 ACAAGAACAACACATCAAGGCCAAGATTTCTAGGGTTTGTGTTCGTACTGAAGCATCCGATACCGGCCTCgtaag
 751 tcctcttaaatctttctttgttgcatgatcgtgtttgagattgcattttgaaatcatcaactcttagatattctt
 826 gagtttctaaacgaagagtggaacatggcatgcatccaggattgtgctatgtctctaaatggtgatatacgacat
 901 gatttcataccaacttagaaaagttaggtttttcgatacaattgattacattaagatgttagttatacgtgtgac
 976 gattaactcaaagaaagaaacaagaaactataatatattttgagcttcatggttatagctttagttataggctgt
 161                                                          L  V  K  D  G  Y
1051 tatttcttatgtttttcaagatttctaaacatgtttgttatttttttgttataaacagTTGGTTAAGGATGGATAT
 167 Q  W  R  K  Y  G  Q  K  V  T  R  D  N  P  S  P  R  A  Y  F  K  C  S  H  A
1126 CAATGGAGGAAATATGGACAAAAGGTTACAAGGGACAACCCTTCTCCTAGAGCTTATTTCAAATGCTCTCATGCT
 192 P  S  C  P  V  K  K  K  V
1201 CCAAGCTGCCCAGTCAAAAAGAAGGTgagaagccattgccaaacatcatctaaattttagtcttaagataaagtc
 201                                                       Q  R  S  V  E  D  Q
1276 tatcgatttcgtgttctaatttgcttctttgttaaatcttgttatcattaggtTCAAAGAAGTGTTGAGGATCAA
 208 S  I  L  V  A  T  Y  E  G  E  H  N  H  P  S  Q  S  K  H  E  Q  A  S  S  G
1351 TCAATCTTGGTAGCAACTTATGAAGGAGAGCACAACCATCCAAGCCAATCAAAACACGAACAAGCGAGCTCAGGA
 233 L  N  R  T  V  T  S  T  T  L  G  S  A  S  L  S  S  S  G  P  T  I  T  L  D
1426 TTGAACCGAACCGTCACAAGCACGACTCTCGGTTCAGCTTCTCTAAGCTCATCTGGACCCACAATCACTTTAGAC
 258 L  T  T  P  P  K  T  P  T  N  P  S  D  E  T  K  V  G  G  D  R  R  V  D  T
1501 TTAACCACCCCACCAAAAACACCAACAAACCCCTCGGACGAAACCAAAGTTGGTGGTGACCGAAGAGTTGACACA
 283 P  E  F  Q  Q  F  L  V  D  Q  M  A  S  S  L  T  K  D  P  S  F  K  A  A  L
1576 CCAGAATTTCAACAGTTTTTGGTAGATCAAATGGCATCCTCATTGACCAAAGATCCGAGCTTTAAAGCAGCATTG
 308 A  A  A  I  S  G  R  M  V  Q  Q  N  Q  S  Q  K  W  *  *
1651 GCAGCTGCGATTTCTGGAAGAATGGTTCAACAAAATCAGTCCCAAAAGTGGTAATAGctactagaagccattgat
1726 catgagatcatg
```
