# Supplementary material for: Genome-Wide Identification of WRKY Genes in Artemisia annua: Characterization of a Putative Ortholog of AtWRKY40
Source: Plants (Basel). 2020 Nov 28;9(12):1669. doi: 10.3390/plants9121669 (PMC7761028; doi:10.3390/plants9121669)
Supplement: Supplementary file 1 [file plants-09-01669-s001.zip › Suppl_rev/LegendFigure S1.pdf]

Figure S1: Nucleotide and deduced amino acid sequences of *AaWRKY40* genomic DNA. WRKY domain containing an invariant WRKYGQK sequence is indicated, putative zinc-finger motif is in bold and the C2H2 motif highlighted in red. Putative leucine zipper motif is highlighted in yellow. Coding regions were indicated in capital letters.
